# Treatment options for PNET liver metastases: a systematic review

**DOI:** 10.1186/s12957-018-1446-y

**Published:** 2018-07-14

**Authors:** Giuseppe Nigri, Niccolò Petrucciani, Tarek Debs, Livia Maria Mangogna, Anna Crovetto, Giovanni Moschetta, Raffaello Persechino, Paolo Aurello, Giovanni Ramacciato

**Affiliations:** 1 Department of Medical and Surgical Science and Translational Medicine, St. Andrea Hospital Rome, Sapienza University of Rome, Via di Grottarossa 1035, 00189 Rome, Italy; 2 Digestive Surgery, Hepatobiliopancreatic Surgery and Liver Transplantation, UPEC University, Henri Mondor Hospital, Creteil, France; 3 Department of Digestive Surgery and Liver Transplantation, Nice University Hospital, Nice, France

## Abstract

**Background:**

Pancreatic neuroendocrine tumors (PNETs) are rare pancreatic neoplasms. About 40–80% of patients with PNET are metastatic at presentation, usually involving the liver (40–93%). Liver metastasis represents the most significant prognostic factor. The aim of this study is to present an up-to-date review of treatment options for patients with liver metastases from PNETs.

**Methods:**

A systematic literature search was performed using the PubMed database to identify all pertinent studies published up to May 2018.

**Results:**

The literature search evaluated all the therapeutic options for patients with liver metastases of PNETs, including surgical treatment, loco-regional therapies, and pharmacological treatment. All the different treatment options showed particular indications in different presentations of liver metastases of PNET. Surgery remains the only potentially curative therapeutic option in patients with PNETs and resectable liver metastases, even if relapse rates are high. Efficacy of medical treatment has increased with advances in targeted therapies, such as everolimus and sunitinib, and the introduction of radiolabeled somatostatin analogs. Several techniques for loco-regional control of metastases are available, including chemo- or radioembolization.

**Conclusions:**

Treatment of patients with PNET metastases should be multidisciplinary and must be personalized according to the features of individual patients and tumors.

## Background

Pancreatic neuroendocrine tumors (PNETs) are rare tumors, representing 1.3 to 10.0% of all pancreatic tumors. Annual incidence of PNET is estimated to be 3.65/10,000 people per year [1–3]. Due to the recent widespread use of diagnostic techniques, there is a dramatic increase in the incidence of PNETs [4]. No differences in PNET incidence are reported between men and women. Peak of PNET’s diagnosis occurs between 30 and 60 years [5]. PNETs may be classified as functioning or non-functioning tumors. Functioning PNETs are characterized by secretion of one or more biologically active peptides, inducing specific clinical syndromes. Secreting products include insulin, gastrin, glucagon, somatostatin, and vasoactive intestinal peptide (VIP). Non-functioning PNETs may secrete peptides, such as chromogranin A and neurotensin, and may be asymptomatic [6]. Diagnosis of non-functioning PNETs is usually late for the absence of specific symptoms; therefore, probability of malignancy is higher if compared with functioning PNETs and reported survival is as low as 30% [7]. PNETs are also characterized by the expression of somatostatin receptors. They may be a component of several syndromes, such as Von Hippel-Lindau syndrome, multiple endocrine neoplasia syndromes, or neurofibromatosis type I [6]. Metastases are detected at diagnosis in about 40–80% of patients with PNET [8]. The more frequent sites are the liver (40–93%), followed by the bone (12–20%) and lungs (8%–10%) [8]. The presence of liver metastases also has a negative impact on the prognosis [9, 10], and the extension of PNET liver metastases is correlated to long-term survival [11, 12]. The development of liver metastasis is related to the histological tumor type and to the site of the primary tumor [13]. Other factors with a strong prognostic impact are as follows: size of the primary tumor, mitotic index, vascular and lymphatic invasion, proliferative activity, metabolite serum concentration, and cellular atypias [14]. Treatment of metastatic PNETs is complex and requires multidisciplinary expertise including medical, interventional, and surgical specialties.

Furthermore, multidisciplinary management of metastatic PNETs is in constant evolution. Therefore, it is important to periodically review the recent acquisition, to provide up-to-date and comprehensive data to clinicians. This review, based on a systematic literature search, aims to discuss metastatic PNET’s management from clinical, biochemical, and radiological diagnosis to treatment, focusing on all treatment possibilities in a multidisciplinary approach.

## Methods

### Search strategy and study selection

A systematic literature search was performed using the PubMed database, in order to identify all studies published up to May 2018 reporting data on patients treated for liver metastases from pancreatic neuroendocrine tumors (PNETs) undergoing surgical treatment, including liver resection or liver transplantation, interventional procedures, or medical treatment. The following MeSH search terms were used: “liver” OR “hepatic,” “metastasis OR metastases,” and “pancreatic neuroendocrine tumor” OR “PNET.” The “related articles” function was used to broaden the search, and all the abstracts and citations of all returned studies were reviewed. The full text was examined, in case of any doubt after reading the article’s abstract. Non-English language studies were excluded. Two authors (NP, LM) examined the articles to establish the inclusion in this review.

## Results

### Search results

**Fig. 1:**
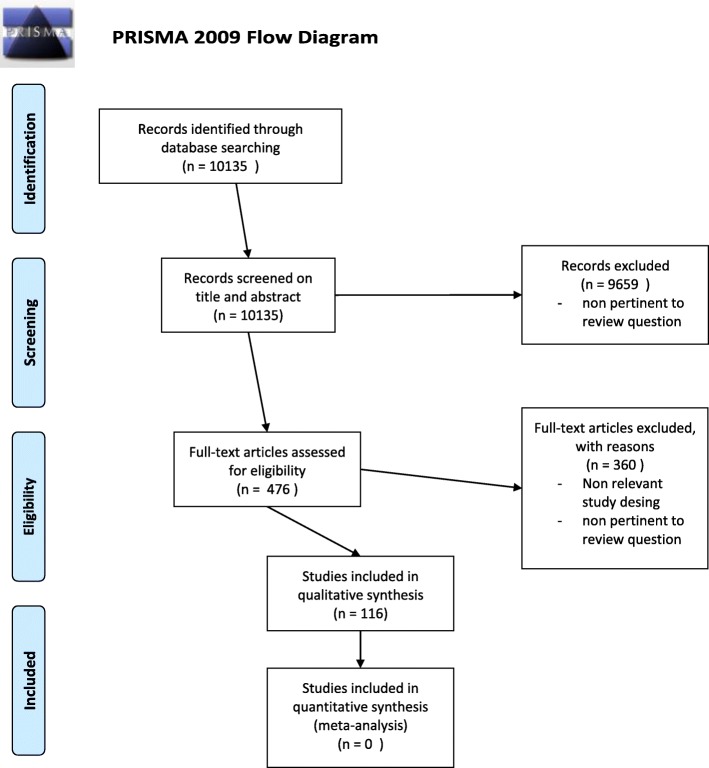
PRISMA diagram showing the systematic search results

Initial search retrieved 10,135 articles. Titles and abstract were analyzed to identify 476 relevant publications. Of them, 116 articles were retained to review the current literature on this topic [1–116]. PRISMA flow diagram is showed in Fig. 1. The majority of them were observational studies. Meta-analyses and review were the second most represented group.

#### Diagnosis and staging

The main factors determining the clinical manifestation of PNET liver metastases are the liver tumor load and the degree of endocrine activity. Usually, patients may remain asymptomatic for a long time. Development of carcinoid syndrome is possible, such as abdominal pain or discomfort. Liver malfunction or failure is a rare occurrence, even in the case of extensive liver involvement [15].

Diagnosis is done on the basis of biochemical laboratory examinations, including specific tumor markers, and on radiological imaging.

Plasmatic chromogranin A is a widely accepted tumor marker, used for diagnostic and prognostic purposes and to evaluate the response to treatment. Its specificity and sensitivity depend on tumor type and volume [16]. 5-Hydroxyindoleacetic acid is a urinary metabolite of serotonin, which may be increased in patients with metastatic PNET, and it is used for diagnosis and follow-up [17]. Other biochemical markers are less used in clinical practice, including urine serotonin, synaptophysin, neuron-specific enolase, parathyroid hormone-related protein, calcitonin, pancreatic polypeptide, and human chorionic gonadotropin [18]. On the other hand, functioning tumors secrete hormones related to a specific clinical syndrome, such as insulin (insulinoma), glucagon (glucagonoma, confirmed by serum glucagon level > 1000 pg/mL), gastrin (elevated serum gastrin and gastric acids), and vasoactive intestinal polypeptide (vipoma, VIP values > 200 pg/mL) [19–21].

Somatostatin receptor scintigraphy is frequently used for PNET imaging. Advantages include the acquisition of whole-body images with possible visualization of the primary tumor and metastases, and the possibility to identify the patients who are candidates for somatostatin receptor-based radiotherapy [22].

Positron emission tomography (PET) with DOTATOC or DOTANOC associated with the positron emitter Gallium 68 allows even better sensitivity (up to 30% higher than standard imaging) [23].

CT scan has wide diffusion and is associated with sensitivity rates up to 94–100% [24], especially if combined with PET [25]. Magnetic resonance imaging is also used in the staging and evaluation of disease progression, for its ability to detect lesions in the liver, combined with reduction of excessive radiation burden [26].

Several staging systems exist for PNET classification. The WHO, European Neuroendocrine Tumor Society (ENETS), and American Joint Committee on Cancer (AJCC) have proposed each a staging system [27–29]. The WHO classification is based on cellular proliferation (measured as mitotic count and Ki-67 expression), as shown in Table 1 [27]. The ENETS staging system (Table 2) is based on TNM classification [28], and the AJCC staging system (reported in Table 3) is developed from the TNM staging system for pancreatic adenocarcinoma [29].

**Table 1:** WHO grading system for PNETs [27]

	Grade 1 (G1)	Grade 2 (G2)	Grade 3 (G3)
Ki-67 index	< 3%	3–20%	> 20%
Mitotic count	< 2/10 HPF	2–20/10 HPF	> 20/10 HPF
Differentiation	Well differentiated	Moderately differentiated	Poorly differentiated

*WHO* World Health Organization, *PNETs* pancreatic neuroendocrine tumors, *HPF* high-power field

**Table 2:** ENETS staging system for PNET [28]

Stage	T	N	M
I	T1	N0	M0
IIA	T2	N0	M0
IIB	T3	N0	M0
IIIA	T4	N0	M0
IIIB	Any T	N1	M0
IV	Any T	Any N	M1

*ENETS* European Neuroendocrine Tumor Society, *PNET* pancreatic neuroendocrine tumors, *T1* tumors < 2 cm limited to the pancreas, *T2* 2–4 cm limited to the pancreas, *T3* > 4 cm limited to the pancreas or invading the duodenum or common bile duct, *T4* tumor invading adjacent structures or large vessels, *N0* no regional lymph node metastases, *N1* regional lymph node metastases, *M0* no distant metastases, *M1* distant metastases

**Table 3:** AJCC staging system for PNETs (7th edition, 2010) [29]

Stage	T	N	M
0	Tis	N0	M0
IA	T1	N0	M0
IB	T2	N0	M0
IIA	T3	N0	M0
IIB	T1–3	N1	M0
III	T4	Any N	M0
IV	Any T	Any N	M1

*AJCC* American Joint Committee on Cancer, *PNET* pancreatic neuroendocrine tumors, *T1* < 2 cm limited to the pancreas, *T2* > 2 cm limited to the pancreas, *T3* tumor extends beyond the pancreas but not involving the celiac axis or SMA, *T4* tumor involves celiac axis or SMA, *N0* no regional lymph node metastases, *N1* regional lymph node metastases, *M0* no distant metastases, *M1* distant metastases

#### Treatment options for metastatic PNETs

The therapeutic options for patients with liver metastases from pancreatic neuroendocrine tumors include surgical treatment, loco-regional therapies, and pharmacological treatment. The decision of the treatment strategy is based on the analysis of patient performance status and comorbidities, on accurate tumor staging, and on evaluation of prognostic factors. Surgery represents the only potentially curative therapy when the disease is completely resectable. In patients with advanced and unresectable disease, however, the therapeutic goal is lengthening survival with the best possible quality of life and palliation of symptoms, using a multidisciplinary approach.

### Liver surgery for pancreatic neuroendocrine liver metastases

Surgery remains the treatment of choice in selected patients with PNETs and resectable liver metastases, because it may provide cure. Liver resection for neuroendocrine metastases is associated to long-term survival advantages and disease control [30, 31]. Surgery may have either curative intent, when complete resection is possible, or palliative intent, when the majority of the tumor burden is removed to control the symptoms of the disease. Due to the rarity of the disease, the majority of published articles on surgical treatments of liver metastases from neuroendocrine tumors report data on neuroendocrine metastases from several primary sites (e.g., GEP-NET metastases).

### Potentially curative surgery

Potentially curative surgery is possible in only 10–25% of patients with liver metastases [32]. Bilobar metastases may be treated with two-step resections, and preoperative portal vein embolization may be used to induce hypertrophy of the left liver lobe, as in colorectal liver metastases [33]. Concurrent or staged resection of the primary lesion and liver metastases may be considered, if surgery can remove most of the metastatic tumor volume (> 90%). Criteria helping to select patients for surgery include the presence of well-differentiated G1/G2 tumors, absence of distant lymph node metastasis, absence of extrahepatic metastasis, absence of diffuse peritoneal metastasis, and absence of right cardiac dysfunction [34].

Unfortunately, tumor relapse in the first 2 years after resection is reported in the majority of patients [35], and a relapse rate of up to 80% at 5 years has been shown [36–50]. Despite the elevated percentage of tumor recurrence, 5-year survival rates approach 85%, which is in favor of an aggressive surgical approach. Morbidity and mortality of liver resection are acceptable with the advancement in preoperative management and surgical techniques, and are comparable to liver resection for other diseases [36–51].

No randomized trials have compared the results of liver resection to other non-surgical treatments for PNET liver metastases [52]. However, retrospective comparisons of the outcomes of patients treated with medical therapies or palliative care or surgery highlight the advantages of surgical treatment. Survival outcomes of curative surgery are better than those of loco-regional therapies, such as liver chemoembolization, as reported by Elias et al., detecting a 5-year survival rate of 71% for 47 patients who underwent partial hepatectomy versus 31% for 65 patients treated with chemoembolization [41]. Furthermore, Tao et al. demonstrated that debulking surgery improves the effect of the subsequent loco-regional treatment [49].

The presence of a single liver metastasis is associated with better survival, as shown by Frilling et al. [32]. In cases of synchronous metastases, simultaneous resection of the primitive tumor and hepatectomy has been reported, with acceptable postoperative morbi-mortality. Sarmiento et al. treated 23 patients who underwent synchronous pancreatic and liver resection. Postoperative mortality was 0%, the major complication rate 18%, and the 5-year survival was as high as 71% [42]. Bonney et al. showed comparable results, with morbidity of 25%, one death in the postoperative period, and a 5-year survival of 70% [48].

#### Cytoreductive surgery

Cytoreductive surgery in patients with PNET liver metastases aims to increase survival, control symptoms, and improve quality of life. Cytoreductive liver resections are indicated in patients with symptoms not controllable with medical or hormonal treatment. It consists of resection of more than 90% of the tumor mass [53, 54]. Recently, Morgan et al. proposed a threshold of > 70%, with the argument that postoperative results are comparable between debulking > 70, > 90, and 100% [55]. Reduction in tumor volume may reduce the immunosuppressive effects of the tumor and decrease the probability of development of further metastases. Surgical debulking is efficient for the symptoms in the majority of patients with functioning PNETs [44–47, 56]. Combined approaches including aggressive surgical resection, ablative therapies, and chemotherapy may be employed to obtain cytoreduction of the tumor [57].

Symptomatic benefits are achieved in 80–90% of patients submitted to curative liver resections [12, 42, 54]. The mean duration of the response to the surgical debulking is correlated with the amount of tumor removed and to the normalization of tumor markers [12]. Recurrence of symptoms occurs in the first 5 years after surgery in the majority of patients [42]. Reported rates of complications and mortality are considered acceptable [12, 42, 44–46, 58–60]. If > 75% of the liver parenchyma is involved, prognosis is considered unfavorable and surgical treatment should be avoided [43].

### Liver transplantation

Liver transplantation represents a potentially curative treatment for liver metastases from PNETs. Early results are promising, and future development of this strategy is possible. Orthotropic liver transplantation (OLT) has been proposed for PNETs for two reasons: the less-aggressive biological behavior of neuroendocrine metastases compared to other metastases and the low percentage of patients with PNET liver metastases candidates for R0 liver resections [8, 61]. However, this indication is restricted because of the lack of donors and the perplexity in allocation of organs to oncological patients. Furthermore, the first studies on liver transplantation for metastatic neuroendocrine tumors were not concordant and reported mediocre results. This was due in part to the lack of valid and homogeneous selection criteria [61–67].

Liver transplantation is considered reasonable if expected overall survival is more than 70% at 5 years and disease-free survival is more than 50% [8]. The best candidates in this setting are young patients (< 50 years old), with no extrahepatic lesions, well-differentiated tumors, and low levels of Ki-67. Mazzaferro et al. proposed the following inclusion criteria [8]: diagnosis of low-grade NET confirmed by histological examination (with low expression of Ki-67), location of the primary tumor in an anatomic area tributary to the portal vein, primary tumor already resected with clear margins, < 50% of liver involvement, stable disease during 6 months before OLT, and age < 55 years. Recently, a comprehensive review showed encouraging 5-year survival after OLT for NET, but a high recurrence rate [68]. So at present, liver transplantation does not represent routine care in this setting and is considered investigational and allowed in the setting of clinical studies [69].

Another debated point is the indication for primary tumor resection in patients with unresectable metastatic disease. Recent retrospective studies [70] and a meta-analysis showed that the palliative resection of the primary tumor in patients with PNETs and unresectable liver metastases may increase long-term survival. The meta-analysis by Zhou and colleagues included 10 studies, with a total of 1226 patients undergoing primary tumor resection and 1623 patients who did not have surgery [71]. The results of the meta-analysis showed a significantly longer survival in patients who had surgical resection of the primary tumor (at 5 years, 35.7–83% surviving patients in the surgical group versus 5.4–50% in the non-surgical group) [71].

#### Liver-directed therapies

Liver-directed therapies used to treat PNET metastases include radiofrequency ablation (RFA), cryoablation, alkalization, transarterial embolization (TAE), and transarterial chemoembolization (TACE) [72].

### Ablative therapies

RFA is a safe technique, generally used to treat unresectable metastases smaller than 5 cm. Associated morbidity is low and mainly consists in bleeding and abscess formation [73]. RFA is effective to treat symptoms related to liver metastases and hormone secretion, even if the tumor size represents a limiting factor. RFA is less useful for tumors > 5 cm, even if repeat ablation sessions are possible [37]. The location of the lesion should be considered, because RFA may be contraindicated for liver metastases near to vital structures or at the liver surface. Cryotherapy is another suitable option, and percutaneous ethanol injection is an alternative in cases where tumors are close to vital structures or vessels [74].

### Hepatic arterial embolization

The rationale of hepatic transarterial embolization is that neuroendocrine metastases receive most of their blood supply from the hepatic artery, whereas normal liver parenchyma gets 75% of its blood supply from the portal vein flow [75]. Both TAE and TACE effectively reduce tumor size and improve patients’ symptoms. No randomized studies comparing the two techniques have been published nor studies comparing embolization techniques with cytoreductive surgery in the palliative segment. Embolization is not associated with risks of tumor dissemination (this is an advantage compared to RFA). During TAE, embolization is performed using lipiodol, gem foam particles, polyvinyl alcohol foam, or bland microspheres, whereas for TACE, chemotherapeutic agents are added, leading to an intra-tumoral drug concentration over 20 times greater than those obtained with systemic administration. Furthermore, both provoke tumor ischemia. Commonly used drugs are doxorubicin, melphalan, and streptozocin. Minor side effects of the procedure are fever, leukocytosis, abdominal pain, and liver cytolysis.

Morbidity rate is low, even if serious complications, such as liver abscess, gallbladder necrosis, bowel ischemia, pleural effusion, and hepatic failure, have been reported [76]. Tumor response is objectivized in 25–86% of cases, and the duration of the response ranges from 6 to 45 months [77–79]. In a recent series, clinical improvement and tumor response were observed in 95% of patients, with median time to tumor progression of 14 ± 16 months and median overall survival of 22 ± 18 months [80].

Both TAE and TACE are considered for palliation in unresectable tumors, especially for functioning tumors with symptoms not controlled by medical therapy. Contraindications of TAE and TACE include portal vein occlusion, insufficient liver reserve, and poor performance status. In patients with previous pancreaticoduodenectomy, transarterial therapies are generally contraindicated, due to higher risks of post-procedure morbidity. Liver-directed therapies may also be proposed in patients with extrahepatic metastases to control liver disease and symptoms [80–83].

Selective internal radiotherapy (SIRT) consists of embolization with 90Yttrium microsphere, a beta-emitter that results in tissue penetration of 2.5 mm. Published data on SIRT show a response rate of 55% and stabilization of the disease in 32% of cases [84–87]. More recently, an overall disease control rate of 88.9% at 3 months after therapy has been demonstrated, confirming its effectiveness in treating unresectable PNET liver metastases [30]. SIRT is contraindicated in cases of aberrant vessels with shunt to the gastrointestinal tract, compromised portal veins, and inadequate liver functional reserve to avoid potentially serious complications.

### Medical therapy

Medical therapy is indicated for advanced unresectable PNETs and includes drugs acting on hormone receptors, conventional chemotherapy, and molecular target therapy [88].

Somatostatin analogs (SSAs) act on somatostatin receptors and are effective in controlling hormonal secretion and tumor growth. Either functioning or non-functioning PNETs express at least one of the five subtypes of somatostatin receptor (SSTR). Different SSAs have specific affinity for different SSTRs [89]. Octreotide and lanreotide have high affinity for SSTR2 and bind to SSTR5, whereas the recent analog pasireotide binds with high affinity to SSTR1, SSTR2, SSTR3, and SSTR5 [89, 90]. Several studies and a randomized controlled trial advocate the use of SSAs to control tumor growth and symptoms in this setting [89, 91, 92]. The randomized controlled trial by Rinke et al. demonstrated that long-acting octreotide is efficacious on both functioning and non-functioning tumors, with a 66.7% reduction in the risk of disease progression in treated patients compared to patients taking a placebo [92]. However, these results referred to a specific setting of patients with limited liver involvement (≤ 10%) and already resected primary tumors. Further randomized trials to confirm these data in other patient categories are needed. The CLARINET (Controlled Study on Lanreotide Antiproliferative Response in NETs) study is an ongoing trial, which aims to evaluate the efficacy of lanreotide in patients with well or moderately differentiated, non-functioning NETs with Ki-67% expression < 10% [93]. A number of other previous studies have advocated for the efficacy of SSAs on PNETs, with tumor stabilization reported in 40–80% of patients and objective tumor response (demonstrated by reduction of tumor volume) in about 10% of patients [94–96]. If treatment with SSAs at standard dose fails, management options include shortening of SSA administration intervals or augmentation of SSA dosage. For patients with progressing tumors, administration of SSAs every 21 days was compared to administration every 28 days, demonstrating a longer time to progression, better symptom control, and reduction in the serum level of tumor markers in the group with the shorter interval of administration [97]. SSAs are well tolerated and have generally mild side effects. Long-term side effects include gallbladder lithiasis (1%), glucose intolerance or diabetes, and steatorrhea [96].

Alpha-interferon may be associated with somatostatin analogs for palliation or hormonal symptoms, with tumor stabilization occurring in 30–80% of patients. Reduction of tumor volume occurs only in a small percentage of patients [98]. Side effects are frequent and include flu-like symptoms (80–90%), anorexia, weight loss, fatigue, bone marrow or liver toxicity, and autoimmune disorders.

Systemic chemotherapy is only indicated for advanced and unresectable PNETs and may consist in the administration of various cytotoxic agents, such as streptozotocin, cisplatin, dacarbazine, doxorubicin, and 5-fluorouracil [99]. The efficacy of the combination of streptozotocin with 5-fluorouracil (5-FU) and/or epirubicin in treating G1/G2 pNENs has been demonstrated, with a reported objective response rate of 20–45% [100, 101]. Alternative options include temozolomide alone or in combination with capecitabine, leading to a partial response rate of 70%, median progression-free survival (PFS) of 18 months, and 2-year survival of 92% in cases of metastatic, well-differentiated PNETs [102]. For high-grade tumors with poor differentiation, platinum-based regimes are preferred. Response rates of 42–67% have been obtained combining cisplatin and etoposide [103]. Saif et al. suggested the use of capecitabine/temozolomide (CAPTEM) regimen in patients with failure of the previous therapy [104].

### Targeted therapies

Recent advancements in comprehension of the pathogenesis and molecular mechanisms of PNETs have allowed the development and introduction of novel targeted therapies in the clinical practice. The mTOR protein is a serine/threonine kinase, and a key component of a cellular pathway playing an important role in the regulation of cell growth and proliferation. mTOR is upregulated in several tumors, including PNETs [105]. Everolimus is an mTOR inhibitor that has shown efficacy in phase II and phase III studies in patients with PNETs [106]. The RADIANT3 study randomized patients with advanced PNET into two groups: patients receiving everolimus (10 mg per day) (group 1) and patients receiving a placebo (group 2). Patients treated with everolimus had significantly longer PFS (11 versus 4.6 months) than patients receiving placebo [107]. Side effects include stomatitis, rash, fatigue, diarrhea, hyperglycemia, and hematological and pneumological effects.

Sunitinib is an inhibitor of the tyrosine kinases PDGFR, VEGFR-1, VEGFR-2, c-KIT, and FLIT3 [108, 109]. The rationale for its use in the treatment of PNETs is the frequent overexpression of VEGF or VEGFR by these tumors. A phase III study comparing sunitinib to a placebo has shown a response rate of 9.3% and an increased PFS of 11.1 months in the group treated with sunitinib (versus PFS of 5.5 months in the placebo group) [110]. Side effects of sunitinib include diarrhea, nausea, vomiting, asthenia, fatigue, hypertension, and neutropenia. Raymond et al. reported a partial tumor response in 42% of patients and stable disease in 33% of patients after treatment with 37.5 mg/day of sunitinib [110].

Radiolabeled somatostatin analogs represent a new treatment option in patients with strong radiotrace uptake on SRS [111]. Peptide receptor radionuclide therapy (PRRT) with radiolabeled SSAs allows administration of targeted radiotherapy to the tumor tissue and its metastases [112]. The most used radiolabels are 90Yttrium, a high-energy beta-particle emitter, and 177Lutetium, which emits beta particles and gamma rays. Even if complete tumor response is rare with this treatment (0–6%), results are encouraging, with partial tumor regression in 7–37% of patients and stabilization in 42–86% using 90Yttrium-labeled SSAs [112–114]. 177Lutetium octreotate was used on 510 patients, 40% of whom had PNETs, and partial response was observed in 28% of cases, with stabilization of the disease in 35% [115, 116]. PRRT is a promising therapeutic option, even if still investigational.

## Conclusions

Therapeutic options for patients with liver metastases from pancreatic neuroendocrine tumors include surgery, loco-regional therapies, and medical therapies. Surgery represents the only potentially curative treatment and should be proposed for resectable patients, even if relapse rates are high. Efficacy of medical treatment has increased with advances in targeted therapies, such as everolimus and sunitinib, and with the introduction of radiolabeled somatostatin analogs. Several techniques for loco-regional control of metastases are available, including chemo- or radioembolization. Treatment of patients with pancreatic neuroendocrine metastases should be multidisciplinary, must be personalized according to the features of individual patients and tumors, and should take into account all possible options in order to provide the best possible results in terms of survival and quality of life.

## Abbreviations

5-FU: 5-Fluororacil
PFS: Progression-free survival
PNET: Pancreatic neuroendocrine tumor
PRRT: Peptide receptor radionuclide therapy
RFA: Radiofrequency ablation
SSAs: Somatostatin analogs
SSTR: Somatostatin receptor
TACE: Transarterial chemoembolization
TAE: Transarterial embolization
SIRT: Selective internal radiotherapy
OLT: Orthotropic liver transplantation
